# ICP-MS Measurement of Trace and Rare Earth Elements in Beach Placer-Deposit Soils of Odisha, East Coast of India, to Estimate Natural Enhancement of Elements in the Environment

**DOI:** 10.3390/molecules26247510

**Published:** 2021-12-11

**Authors:** Nimelan Veerasamy, Sarata Kumar Sahoo, Rajamanickam Murugan, Sharayu Kasar, Kazumasa Inoue, Masahiro Fukushi, Thennaarassan Natarajan

**Affiliations:** 1 National Institute of Radiological Sciences, National Institutes for Quantum Sciences and Technology (QST), 4-9-1 Anagawa, Inage-ku, Chiba 263-8555, Japan; nimelanveerasamy@gmail.com (N.V.); murugan.rajamanickam@qst.go.jp (R.M.); kasar.sharayu@qst.go.jp (S.K.); nthennarassan@gmail.com (T.N.); 2 Department of Radiological Sciences, Tokyo Metropolitan University, 7-2-10 Higashiogu, Arakawa-ku, Tokyo 116-8551, Japan; kzminoue@tmu.ac.jp (K.I.); fukushi@tmu.ac.jp (M.F.)

**Keywords:** soils, trace elements, rare earth elements, geoaccumulation index, enrichment factor, ICP-MS

## Abstract

Inductively coupled plasma mass spectrometry (ICP-MS) has been used to measure the concentration of trace and rare earth elements (REEs) in soils. Geochemical certified reference materials such as JLk-1, JB-1, and JB-3 were used for the validation of the analytical method. The measured values were in good agreement with the certified values for all the elements and were within 10% analytical error. Beach placer deposits of soils mainly from Odisha, on the east coast of India, have been selected to study selected trace and rare earth elements (REEs), to estimate enrichment factor (EF) and geoaccumulation index (I_geo_) in the natural environment. Enrichment factor (EF) and geoaccumulation index (I_geo_) results showed that Cr, Mn, Fe, Co, Zn, Y, Zr, Cd and U were significantly enriched, and Th was extremely enriched. The total content of REEs (ƩREEs) ranged from 101.3 to 12,911.3 µg g^−1^, with an average 2431.1 µg g^−1^ which was higher than the average crustal value of ΣREEs. A high concentration of Th and light REEs were strongly correlated, which confirmed soil enrichment with monazite minerals. High ratios of light REEs (LREEs)/heavy REEs (HREEs) with a strong negative Eu anomaly revealed a felsic origin. The comparison of the chondrite normalized REE patterns of soil with hinterland rocks such as granite, charnockite, khondalite and migmatite suggested that enhancement of trace and REEs are of natural origin.

## 1. Introduction

Environmental pollution has pervaded many parts of the world due to anthropogenic activities such as urbanization, exploration, mining of natural resources, industrialization, etc., which has resulted in contamination of trace elements (TEs) and REEs into the environment directly or indirectly [1,2,3]. Natural contents of REEs in soil are highly influenced by their parent materials, weathering and pedogenesis processes [4]. In soil, the enrichment of REEs is mainly controlled by the abundance of REE-bearing minerals such as apatite, allanite, bastnaesite, monazite, xenotime and zircon [5]. There are a few reports showing a gradual increase in REEs in soil by anthropogenic activities [6,7]. The REEs background data could be used as baselines to identify contamination level as well as quantitative risk assessment in soils. Therefore, monitoring of TEs and REEs is essential for the establishment of baselines from the viewpoint of environmental pollution or contamination. Geochemical analyses of natural materials (soils, sand, etc.) are necessary to determine the level of contamination, and to elucidate whether it is from geogenic or anthropogenic sources [8]. Environmental contaminations have been evaluated using two pollution indices such as the enrichment factor (EF) and geoaccumulation index (I_geo_), to identify the degree of contamination in soil and sediments and their origin [9].

Beach placer deposits are formed by sediments produced through weathering and erosion of rocks (i.e., igneous, sedimentary and metamorphic rocks) that are transported by rivers and streams to coastal areas. During these processes heavy minerals (specific gravity, ρ > 2.89 g/cm^3^) such as monazite, ilmenite, zircon, rutile, garnet, and sillimanite are accumulated along the beaches [10]. Monazite [(Ce, La, Nd, Th) PO_4_] is an important heavy mineral containing a high concentration of Th and rare earth elements (REEs), especially light REEs (LREEs) [11,12].

Recently, increasing attention has been paid towards not only environmental radioactivity studies but also to the origin of beach placer deposits in the southwest coast of Sri Lanka [13], Sithonia Peninsula, Greece [14], Calabria, Italy [15], Langkawi, Malaysia [16], Chittagong, Bangladesh [17], and Mandena, Madagascar [18]. Several Indian coastal areas, well-known as high background radiation areas (HBRAs), with beach placer deposits have been investigated; these areas are in Karnataka [19]; Andhra Pradesh [20]; Kerala [21]; Tamil Nadu [22,23,24] and Odisha [25,26,27,28].

The Odisha state is an important littoral state on the eastern coast of India, and the coastal stretch between the Rushikulya river and Gopalpur town is known as the Chhatrapur–Gopalpur beach placer deposit. The total weight percentage of heavy minerals in this beach placer deposit ranges from 2.9 to 20.4%. It includes heavy minerals such as garnet, hornblende, ilmenite, magnetite, monazite, pyroxene, rutile, sillimanite, sphene, tourmaline and zircon [29,30]. Due to the high accumulation of monazites, ilmenites and rutiles minerals in the beach sand, this region has been explored by Indian Rare Earth Limited (IREL) and an extensive exploration process is in progress [10]. Eventually, this will lead to the possibility of anthropogenic contamination in the environment. Therefore, environmental monitoring studies with respect to pollution and contamination are necessary.

In Odisha’s coastal soils, there is a lack of TEs and REEs data of bulk sand and soil in the Chhatrapur–Gopalpur beach placer deposits. The REEs background data could be used as baselines to identify contamination level as well as to conduct a quantitative risk assessment in soils. Therefore, analyses of TEs and REEs in soils have been carried out using inductively coupled plasma mass spectrometry (ICP-MS) to evaluate two pollution indices, the enrichment factor (EF) and geoaccumulation index (I_geo_), to identify the degree of environmental contamination.

(1) To validate analysis of TEs and REEs with certified reference materials using ICP-MS;
(2) Determination of TEs and REEs in Chhatrapur–Gopalpur beach placer-deposit soils;
(3) Estimation of EF and I_geo_ of TEs to evaluate natural enrichment and anthropogenic contamination in soils;
(4) To understand the origin/source of TEs and REEs in beach placer-deposit soils.

## 2. Results and Discussion

### 2.1. Analytical Validation of TEs and REEs

In this study, geochemical certified reference materials (CRMs) such as Japan lake sediment (JLk-1) and Japan basalts (JB-1 and JB-3), supplied by the Geological Survey of Japan, were used to the validate analytical method for TEs and REEs using ICP-MS. The concentrations (µg g^−1^) of TEs such as Cr, Mn, Fe, Co, Ni, Cu, Zn, Rb, Sr, Zr, Cd, Cs, Ba, Pb, Th and U and REEs (Y, La, Ce, Pr, Nd, Sm, Eu, Gd, Tb, Dy, Ho, Er, Tm, Yb, Lu) are given in Table 1. The TEs and REEs results were compared with the certified values of CRMs [31,32]. The recovery of the mean measured values of JLk-1, JB-1 and JB-3 for TEs and REEs ranged from 90 to 110%.

Errors of analysis are represented as standard deviation (SD) which refers to the precision [33]. The accuracy as a relative bias (RB%) of the measurement of TEs and REEs was ≤10%. This states that the reproducibility as a measure of precision of the analytical method is in good agreement with the certified values for TEs and REEs, i.e., within analytical error of 10%. The same method was applied to all soils.

### 2.2. TEs in Beach Placer-Deposit Soils

The mean concentration of TEs (µg g^−1^) of each sample location from the study area are summarized in Table 2. The results showed that the mean concentration of elements in the soils are in the following order: Fe > Mn > Th > Ba > Zr > Y > Cr > Zn > Pb > U > Rb > Co > Sr > Ni > Cu > Cs > Cd.

The Fe (iron) concentration in samples varied from 19,000 to 150,000 µg g^−1^ with an average of 57,508 µg g^−1^, i.e^.^, higher than World Health Organization (WHO) global limit (50,000 µg g^−1^) [34]. The Fe concentration was high in the samples collected from Aryapalli, Boxipalli, Kanamana, Gopalpur and Matikhalo. The Mn concentration varied from 460 to 3700 µg g^−1^ with an average value of 1300 µg g^−1^ and was less than the WHO critical value (2000 µg g^−1^) [34]. However, Aryapalli samples showed Mn concentration more than 2000 µg g^−1^.

Concentration of Th ranged from 35.0 to 900 µg g^−1^ with a mean value of 390 µg g^−1^. The high concentration of Th in the soils is attributed to the presence of monazite minerals. U concentration varied from 1.4 to 53.2 µg g^−1^ with a mean value of 14.6 µg g^−1^. Pb concentration ranged from 16.2 to 65.0 μg g^−1^ with a mean value of 40.0 μg g^−1^. The highest Pb concentration was observed at Aryapalli, however all samples were below global limit 85 μg g^−1^. The presence of Pb in the human body causes damage to bones and organs such as the liver, kidneys, brain, lungs, and central nervous system. Ba concentration varied from 3.4 to 385 µg g^−1^ with a mean value of 142 µg g^−1^. The highest concentration of Ba was observed at Jagnyasala.

Zn concentration varied from 27.0 to 250 µg g^−1^ with a mean value of 103 µg g^−1^. The highest concentration of Zn was observed at Aryapalli, Kanamana, Matikhalo and Venkatraipur. Zr concentration varied from 2.2 to 370 µg g^−1^ with a mean value of 102 µg g^−1^, which was less than the average upper continental crust (UCC) value of 190 µg g^−1^. Cr concentration varied from 35.6 to 180 µg g^−1^ with a mean value of 83 µg g^−1^. The mean concentration was less than the global limit of 150 μg g^−1^. The highest concentration of Cr was observed at Aryapalli. A high concentration of Cr causes skin related diseases. Co concentration varied from 10.4 to 75.0 μg g^−1^ with a mean value of 27.4 μg g^−1^. Ni concentration varied from 1.1 to 24.5 μg g^−1^ with a mean value of 12.0 μg g^−1^. Other trace elements were in very low concentrations—below the recommended global limits.

### 2.3. Enrichment Factor (EF) of TEs in Soil

The EF results of trace elements in soils are given in Table 3. The results showed that Th was extremely enriched in Aryapalli, highly enriched in Boxipalli, significantly enriched in Kanamana, Badaputti, Matikhalo, Gopalpur, Kalipalli, Chhatrapur and Venkatraipur, and moderately enriched in Basanaputi. U was extremely enriched in Aryapalli, highly enriched in Boxipalli, significantly enriched in Kanamana, Matikhalo, Gopalpur, Kalipalli, Chhatrapur, and Venkatraipur and moderately enriched in Badaputti. The extreme enrichment of Th and U in the soils could be explained mainly by the presence of monazite minerals and felsic-source rocks in the study area. There were no anthropogenic activities related to the enrichment of Th and U.

Cr, Mn, Fe, Co, Zn, Y, Zr, and Cd were significantly enriched in Aryapalli samples. Cs has been significantly enriched in Chhatrapur and Boxipalli. Mn, Fe and Co were significantly enriched in Kanamana samples. Cr, Mn and Co were significantly enriched in Gopalpur samples. Cr, Mn, Fe, Cu, Sr, and Cs were significantly enriched in Chattrapur samples. Cr and Co were significantly enriched in Matikhalo samples. Mn and Y were significantly enriched in Boxipalli samples. The significant enrichment of Cr may be due to the mafic-source rock present in the study area. Mn and Fe enrichment may be due to the presence of ilmenite mineral present in the study area.

### 2.4. Geoaccumulation Index (I_geo_) of TEs in Soils

The results of I_geo_ values for the elements in soils are presented in Table 4. Th was extremely enriched in Aryapalli, Boxipalli, Kanamana and Matikhalo, and highly enriched in Gopalpur, Kalipalli and Venkatraipur. Enrichment of Th in Chhatrapur was moderate to high, whereas it was moderately enriched in Badaputti and Basanaputti and slightly enriched in Jagnyasala and Kalyaballi. U is moderately to heavily enriched in Aryapalli and Boxipalli, slightly enriched in Kanamana, Matikhalo, Kalipalli, and Venkatraipur. Pb and Y were slightly enriched in Boxipalli. Mn, Co and Zn were slightly enriched in Aryapalli. The slight enrichment of Pb is due to the mining activities near the Aryapalli and Boxipalli study areas.

### 2.5. Geochemistry of REEs in Soils

The mean concentrations of light and heavy REEs (LREE and HREE) from all samples are given in Table 5 along with descriptive statistics. The mean ∑LREEs (2308.8 µg g^−1^) concentration was about 17 times higher than the UCC value (132.5 µg g^−1^). On the other hand, the mean ∑HREEs (71.2 µg g^−1^) concentration was five times higher than the UCC value (13.9 µg g^−1^). The total concentrations of ∑REEs ranged from 101.3–12911.3 µg g^−1^ with a mean value of 2431.1 µg g^−1^. The mean ∑REEs concentration was 16 times higher than the UCC value (146.4 µg g^−1^) [35].

The enrichment of REEs (µg g^−1^) was in the following order: Ce (1121.5) > La (540.7) > Nd (458.5) > Pr (119.1) > Sm (66.9) > Gd (39.9) > Dy (13.4) > Er (5.4) > Yb (4.3) > Tb (4.2) > Ho (2.0) > Eu (1.9) > Lu (0.9) > Tm (0.9). The REEs concentrations exhibited the same order as for the Oddo-Harkins rule with two exceptions (i.e., depletion of Eu and slight enrichment of Lu). This type of small exception in the order of REE concentrations has been observed in Cuban soils [36]. The REEs concentration in the study area has been arranged in decreasing order as follows: Aryapalli > Boxipalli > Kanamana > Gopalpur > Matikhalo > Chhatrapur > Venkatraipur > Kalipalli > Badaputti > Basanaputti > Kalyaballi > Jagnyasala.

Pearson’s correlation coefficients (significant at the 99% level) were used to understand the relationship between Th, U and REEs. The coefficients are presented in Table 6. The results indicate that there is a stronger correlation in LREEs than HREEs. Th showed a stronger positive correlation with LREEs (R^2^ = 0.64 to 0.90) compared to HREEs (R^2^ = 0.46 to 0.83). This positive correlation between Th and LREEs corroborates that Th is a high-field-strength element and strongly supports the presence of monazite minerals. REEs showed similarities in behaviour including low solubility and immobility during weathering and sedimentation [37]. U also showed a strong positive correlation with all REEs (R^2^ = 0.62 to 0.99).

**Figure 1:**
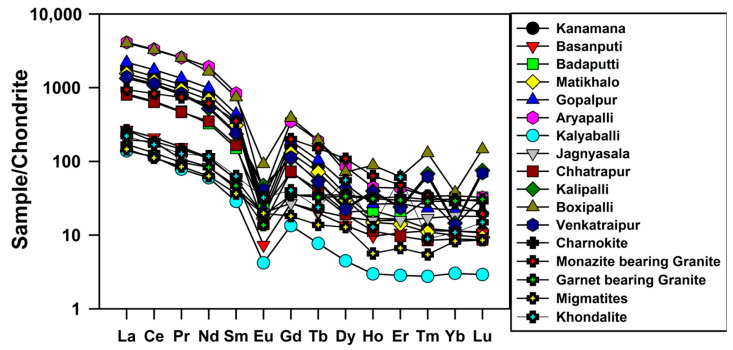
Chondrite normalized REE patterns of soils and hinterland rocks.

In this study, Leedey chondrite values [38] were used for REEs normalization of soils. The chondrite normalized REE patterns of soils are shown in Figure 1. The soils showed enrichment of LREEs and a flat HREEs pattern with negative Eu anomaly. Although the absolute concentrations of REEs in the soils were different, the distribution of chondrite normalized REE patterns of individual samples was remarkably similar. The chondrite normalized REE patterns uniformly showed a high concentration of LREEs and a relatively high concentration of Gd, Tb and Dy in all samples.

The europium (*Eu_A_*) anomaly of the samples was estimated as follows:

(1) $$Eu_{A}=\frac{Eu_{N}}{\sqrt{Sm_{N}\times Gd_{N}}}$$

here, *Sm_N_* and *Gd_N_* are the concentrations of samarium and gadolinium of the bulk soils normalized with respect to the chondrite value.

An Eu anomaly value equal to 1 indicates no anomaly. If the value is >1, there is a positive anomaly and if <1, there is a negative anomaly. All the samples had prominent, negative Eu anomalies (Figure 1). The Eu anomaly values of the soils ranged from 0.06 to 0.78. Similar observations in coastal sediments have been reported in the literature [39,40]. The negative Eu anomaly is a peculiar characteristic of felsic rocks, e.g., granite [41]. The soils had higher LREE/HREE ratios with a strong negative Eu anomaly, which suggested that the soils might have been derived from a felsic source.

The LREEs enrichment and positive correlation of Th in soils confirmed the presence of monazite mineral, and the relatively high concentration of Gd, Tb and Dy might be due to the presence of hornblende, pyroxene and garnet. To confirm the source rocks of the Chhatrapur–Gopalpur beach placer deposits, the REE patterns of various rock types present in the hinterland regions compared with soils are shown in Figure 1. The hinterland rocks comprised charnockite, khondalite, migmatite, monazite-bearing granite and garnet-bearing granite. The REE data on hinterland rocks were mainly granite [42], migmatite and charnockite [43] and khondalite [44]. The chondrite normalized REE patterns of charnockite, khondalite, granulite and granite were plotted to compare them with soil patterns. The obtained chondrite normalized REE patterns of soils were almost same as the chondrite normalized REE patterns of granite, migmatite, khondalite and charnockite. Hence, granite, charnockite, and migmatite might be the major source rocks for monazite and other heavy minerals present in the soils.

### 2.6. Possible Source for TEs and REEs Enrichment in Soils

**Figure 2:**
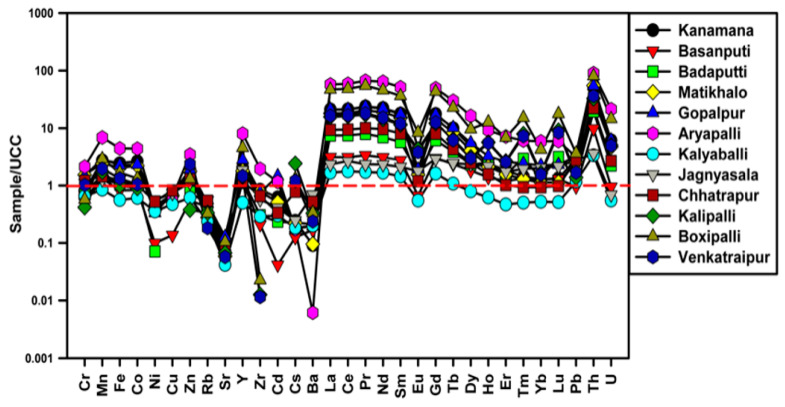
Plot showing UCC-normalized TEs and REEs patterns.

The TEs and REEs in soils were normalized with UCC and plotted in Figure 2. The UCC-normalized multielement diagram showed the enrichment of Mn, Fe, Co, Zn, Y, Pb, Th, U and REEs. Among these, Th and REEs are more enriched. Whereas the other elements were depleted compared to UCC values. The elements’ enrichment values observed from the calculated EF, I_geo_, and UCC normalized patterns were almost similar in the soils.

The EF results show the enrichment of Mn and Fe, which could be due to presence of a solid solution form of ilmenite (Fe, Mn, Ti)O_3_. These minerals are manganiferous end members of the solid solution series [45]. The EF and I_geo_ results showed high enrichment of Th as well as high concentration of REEs, which could be assigned to the presence of monazite minerals in the soils. Therefore, it indicated that the enrichment of high Th, U and REEs are from natural origin and without involvement of any anthropogenic activities.

## 3. Materials and Methods

### 3.1. Study Area

**Figure 3:**
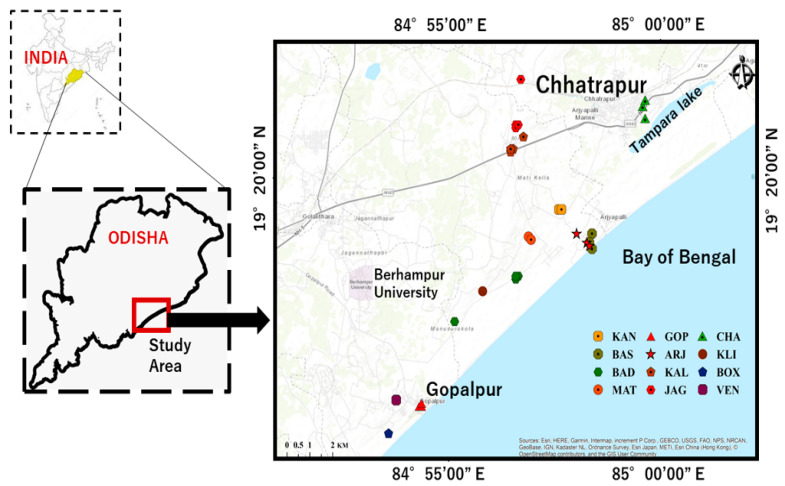
Map showing geographical locations of soils and ambient dose rates. (KAN—Kanamana; BAS—Basanaputti; BAD—Badaputti; MAT—Matikhalo; GOP—Gopalpur; ARJ—Aryapalli; KAL—Kalyaballi; JAG—Jagynasala; CHA—Chhatrapur; KLI—Kalipalli; BOX—Boxipalli; VEN—Venkatraipur). This map was prepared using Arc GIS 10.1. and enhanced using Coral draw software.

The Chhatrapur–Gopalpur beach placer deposits are in the Ganjam district of Odisha, India. These areas extend 20 km length from Chhatrapur City in the north to Gopalpur Town in the south (19° 15′–19° 35′ N Lat; 84° 50′–85° 00′ E Long) with an average width of more than 2 km. A map showing the locations of sampling stations is given in Figure 3. The Bay of Bengal is on the south-eastern side of the study area, and the Eastern Ghats Mobile Belt (EGMB) is on the north and north-western sides. The main drainage system of this area is the Rushikulya River, which originates from the highlands of the EGMB and flows to the sea near Chhatrapur City. Many streams originate in the nearby coastal hills which are ephemeral in nature and could be major suppliers of sediments [46].

The Chhatrapur–Gopalpur beach placer deposits overlay high-grade granulite and intrusive rocks of the EMGB. The major litho-units of the EMGB are khondalite, charnockite and migmatite. The heavy minerals in the beach placers are ilmenite (39.01 mt), garnet (29.40 mt), sillimanite (17.91 mt), rutile (1.81 mt), zircon (1.33 mt) and monazite (1.13 mt) [11]. This study area has paleo dunes, sand bars, planted beach ridges, and red soils with heavy minerals [29].

### 3.2. Sampling and Sample Preparation

Soil samples were collected from a surface layer (0–10 cm depth) using a Daiki soil sampler. At each sampling point, five samples were taken from an area of about 1 m^2^, and these samples were mixed to form a composite sample. Before collection, stones, grass, litter, roots, and shoots were removed from the surface layer. The sampling site selection was based on ambient dose rate, measured using a CsI (Tl) scintillation survey meter (PDR-101, Hitachi-Aloka Medical, Ltd., Tokyo, Japan). Three composite samples were obtained from each sampling location. Approximately 2 kg of each of the 36 composite samples were collected from corresponding 12 sampling locations of the study area. These were brought to the laboratory and air-dried at room temperature. After manually removing remaining roots, shoots, and stones, they were sieved using a 2 mm mesh sieve. The sieved samples were oven-dried at 110 °C for 24 h. Then, all samples were pulverized using a ball mill to less than 150 µm in size prior to chemical decomposition.

### 3.3. Measurement of Trace Elelements and REEs

About 250 mg of homogenized soil samples were ashed in a muffle furnace (KDF-S70, Kyoto, Japan) to decompose organic matter. In the furnace, temperature was increased sequentially as follows: 100 °C for 2 h, 200 °C for 3 h and 600 °C for 5 h. After that it was allowed to cool down for a further 7 h. The furnace-dried samples were chemically digested using a microwave (Milestone MLS 1200 Mega, Sorisole, B.G., Italy) in a closed PTFE pressure vessel with a mixture of concentrated HNO_3_, HF, HClO_4_ and HCl (Tama Pure Chemical Industries, Kawasaki, Japan). The microwave digestion was carried out in two steps. In step one, a mixture of concentrated HNO_3_ (3 mL), HF (2 mL) and HClO_4_ (0.5 mL) was added, and the digestion method was operated at a temperature of 80 °C and 600 W power for 2 h, including cooling time. In step two, a mixture of HNO_3_ (3 mL) and HF (1 mL) was added and the method was similar to step one. The microwave-digested solution was followed by open digestion using aqua regia (HCl (3 mL): HNO_3_ (1 mL)) at 200 °C for 2 h in a clean fume hood. After complete evaporation of aqua regia, the residue was dissolved in 10 mL of 6 M HCl and dried completely. Finally, the sample solution was prepared in 20 mL of 3% HNO_3_. An experimental blank solution was also processed in the same way.

An internal standard Rh was spiked into each diluted sample to correct the signal attenuation due to the presence of various constituents in the samples (matrix effect) as well as for possible changes during ICP-MS measurement. The concentrations of TEs (Al, Cr, Mn, Fe, Co, Ni, Cu, Zn, Rb, Sr, Cd, Ba, Pb, Th and U) and REEs (Y, La, Ce, Pr, Nd, Sm, Eu, Gd, Tb, Dy, Ho, Er, Tm, Yb and Lu) in the decomposed samples were determined using an ICP-MS system (Agilent Technologies 8800 Triple Quad, Tokyo, Japan). The ICP-MS instrument was equipped with a MicroMist nebulizer and a Peltier-cooled (2 °C) Scott-type spray chamber for sample introduction. There was also an octopole-based collision/reaction cell, located between two quadrupole analyzers. The instrument was operated in a gas mode with He (flowing at 5 mL/min) to remove polyatomic ion interferences in case of multielement analysis. The analytical procedure has been described elsewhere [33]. The ICP-MS detection limit was calculated as three times the standard deviation of the calibration blank measurements (*n* = 5). The detection limits varied from (0.03 to 0.2) × 10^−6^ µg g^−1^ for all elements.

### 3.4. Pollution Indices

The pollution indices are an objective tool to assess the enrichment of elements in soils. The individual indices were used to obtain information on the level of soil pollution using each element’s analysed data. The complex indices were used to determine the total pollution of an area. The simultaneous use of several indicators allows us to assess the pollution of soil with elements more accurately [47]. The pollution indices, namely enrichment factor (EF), and geoaccumulation index (I_geo_), were used in the present study to evaluate the level of contamination in the soils.

In the present study, the EF was used to evaluate the influences of natural enrichment and anthropogenic contamination in the soils with respect to the reference sample in the study area. The EF was calculated using Equation (2).

(2) $$EF_{\left(El\right)}=\frac{\frac{Conc{\left(El\right)}_{sample}}{Conc{\left(X\right)}_{sample}}}{\frac{Conc{\left(El\right)}_{Ref\;Sample}}{Conc{\left(X\right)}_{Ref\;Sample}}}\;\;$$

here, “*El*” is the element under consideration, “*Conc*” is concentration (µg g^−1^), and “*X*” stands for the reference element [48]. The subscripts “*sample*” and “*Ref. sample*” indicate their respective concentrations.

The normalized EF has been applied to differentiate element sources as anthropogenic or natural [49]. The TEs, Th, U, and Al average values of Jagnyasala samples (Table 2) are used as a reference sample for this calculation. In general, the EF was classified as unpolluted (EF < 2); moderate (2 < EF < 5); significant (5 < EF < 20); very high (20 < EF < 40), and extremely high (EF > 40). Soil samples’ contamination level can be categorized based on the enrichment factor.

The *I_geo_* was calculated using Equation (2), proposed by [50]. The I_geo_ classification was used to determine the level of contamination.

(3) $$I_{geo}=Log_{2}\left[C_{i}/1.5B_{i}\right]\;$$

here, *C_i_* is the element concentration in soil, *B_i_* is the geochemical background value of an element (average value of UCC) and 1.5 is the coefficient of variation attributed to natural rock.

The geochemical background values of Cr, Mn, Fe, Co, Ni, Cu, Zn, Rb, Sr, Y, Zr, Cd, Cs, Ba, La, Ce, Pr, Nd, Sm, Eu, Gd, Tb, Dy, Ho, Er, Tm, Yb, Lu, Pb, Th, and U are 92, 774, 79344, 17.3, 47, 28, 67, 84, 320, 21, 193, 0.09, 4.9, 624, 31, 63, 7.1, 27, 4.7, 1, 4, 0.7, 3.9, 0.83, 2.3, 0.3, 2, 0.31, 17, 10.5, and 2.7 µg g^−1^, respectively [35]. There are seven classifications in this category. These are: uncontaminated (*I_geo_* ≤ 0; Class 0), uncontaminated to moderately contaminated (*I_geo_* 0–1; Class 1), moderately contaminated (*I_geo_* 1–2; Class 2), moderately to strongly contaminated (*I_geo_* 2–3; Class 3), strongly contaminated (*I_geo_* 3–4; Class 4), strongly to extremely contaminated (*I_geo_* 4–5; Class 5), and extremely contaminated (*I_geo_* ≥ 5; Class 6). In this study, the contamination is considered as enrichment.

## 4. Conclusions

In this study, the concentration of TEs and REEs in Odisha beach placer-deposit soils were determined. EF values showed extreme enrichment of Th, U and significant enrichment of Cr, Mn, Fe, Co, Zn, Y, Zr, Cd and Cu. The extreme enrichment of Th was followed by U, Mn, Co, and Zn, Pb and Y, a slight enrichment was observed in the I_geo_ results. The enrichment of Mn, Fe, Co, Zn, Y, Pb, U, Th, and REEs was observed in the multielement diagram normalized with UCC values. The high concentrations of Fe and Mn were due to the presence of ilmenite heavy mineral, U was due to the presence of zircon, and the enrichment of LREEs and Th was due to the presence of monazite in the soils. Investigation of the REEs geochemistry revealed that the sources of monazite and other heavy minerals might have been derived from charnockite, migmatite, khondalite and granite rocks of the EGMB. The enrichment of elements in the soils is natural in origin. Consequently, the present data in this study will be used as a baseline for future monitoring of TEs and REEs levels in Chhatrapur–Gopalpur beach placer-deposits soils, where it is expected that substantial economic exploration into heavy minerals will occur in the coming decades.

## Figures and Tables

**Table 1:** Analytical results of TEs and REEs (µg g^−1^) for JLk-1, JB-1 and JB-3.

Elements	JLk-1				JB-1				JB-3			
	Mean (µg g^−1^)	SD	CV (µg g^−1^)	Recovery (%)	Mean (µg g^−1^)	SD	CV (µg g^−1^)	Recovery (%)	Mean (µg g^−1^)	SD	CV (µg g^−1^)	Recovery (%)
Cr	71.4	0.3	69	103	433.3	1.3	425	102	55.3	0.2	58.1	95
Mn	2358	7	2092	103	1217	4	1200	101	1453	3	1400	104
Fe	47,538	94	46,738	102	63,605	362	62,900	101	82,355	70	82,700	100
Co	18.5	0.1	18	103	37.9	0.2	38.2	99	34.9	0.2	34.3	102
Ni	38.0	0.2	35	109	133.4	1.2	133	100	36.0	0.4	36.2	99
Cu	69.1	0.3	62.9	110	51.3	0.3	55.1	93	183.9	0.7	194	95
Zn	166.2	0.3	152	109	83.7	4.6	85.2	98	108.0	2.2	100	108
Rb	160.6	0.6	147	109	40.6	0.8	41.3	98	15.6	0.2	15.1	103
Sr	68.8	1.6	67.5	102	431.9	0.1	444	97	424.7	5.3	403	105
Y	43.1	0.2	40	108	23.5	0.7	24.3	97	25.9	0.2	26.9	96
Zr	125.1	0.7	137	91	135.8	1.0	141	96	94.1	1.0	97.8	96
Cd	0.61	0.06	0.57	107	0.12	0.01	0.11	109	0.082	0.008	0.081	101
Cs	10.7	0.2	10.9	98	1.3	0.1	1.2	108	0.92	0.09	0.94	98
Ba	595.7	4.7	574	104	518.2	0.5	493	105	254.5	3.0	245	104
Pb	46.7	0.4	43.7	107	10.0	0.2	10	100	5.6	0.2	5.6	100
La	44.0	0.2	40.6	108	37.7	0.2	38.6	98	8.3	0.1	8.8	94
Ce	89.8	0.6	87.9	102	65.5	0.6	67.8	97	21.0	0.3	21.5	98
Pr	9.3	0.2	8.5	109	6.9	0.3	7	99	3.1	0.3	3.1	100
Nd	35.0	0.3	35.7	98	25.9	0.4	26.8	97	15.1	0.2	15.6	97
Sm	8.1	0.2	7.9	103	5.0	0.2	5.1	98	4.3	0.1	4.3	100
Eu	1.3	0.1	1.3	100	1.6	0.1	1.5	107	1.3	0.1	1.3	100
Gd	6.3	0.3	6	105	4.5	0.4	4.9	90	4.7	0.3	4.7	100
Tb	1.2	0.1	1.2	100	0.80	0.02	0.82	98	0.80	0.03	0.73	110
Dy	6.2	0.2	6.6	94	4.0	0.2	4.1	98	4.6	0.1	4.5	102
Ho	1.1	0.1	1.1	100	0.80	0.04	0.79	101	0.80	0.07	0.80	100
Er	3.8	0.2	3.6	106	2.3	0.1	2.3	100	2.6	0.2	2.5	104
Tm	0.57	0.01	0.53	108	0.38	0.02	0.35	109	0.46	0.02	0.42	110
Yb	3.9	0.2	4	98	2.1	0.1	2.1	100	2.5	0.2	2.6	96
Lu	0.55	0.02	0.57	96	0.31	0.02	0.31	100	0.41	0.01	0.39	105
Th	19.4	0.4	19.5	99	9.3	0.3	9.3	100	1.3	0.1	1.3	100
U	3.7	0.1	3.8	97	1.7	0.1	1.7	100	0.50	0.01	0.48	104

**Table 2:** Mean concentration (µg g^−1^) of TEs in soils.

Elements	Aryapalli	Boxipalli	Kanamana	Gopalpur	Chhatrapur	Matikhalo	Kalipalli	Venkatraipur	Badaputti	Basanaputi	Jagnyasala	Kalyaballi
Cr	180.0 ± 20	48.6 ± 4.5	68.4 ± 6.5	125.0 ± 12	103.0 ± 11	130.0 ± 15	35.6 ± 3.4	87.1 ± 7.9	61.8 ± 6.3	51.4 ± 5.1	43.1 ± 3.8	55.3 ± 5.3
Mn	3700 ± 43	1600 ± 18	1500 ± 16	1600 ± 18	780 ± 9	1300 ± 14	940 ± 10	1060 ± 11	830 ± 9	700 ± 8	690 ± 7	460 ± 5
Fe	1.5 × 10^5^ ± 1533	58,000 ± 777	85,000 ± 870	77,700 ± 787	43,000 ± 428	70,000 ± 661	34,000 ± 348	46,000 ± 458	36,000 ± 359	28,000 ± 261	37,000 ± 362	19,000 ± 189
Co	75.0 ± 7.3	22.5 ± 2.9	44.9 ± 4.6	39.2 ± 4.2	17.4 ± 1.7	37.6 ± 3.2	15.3 ± 1.4	17.8 ± 1.6	17.4 ± 1.6	14.3 ± 1.3	16.8 ± 1.7	10.4 ± 1.0
Ni	17.3 ± 1.6	1.1 ± 0.1	19.5 ± 1.9	14.7 ± 1.5	23.2 ± 2.3	16.6 ± 1.5	1.2 ± 0.1	1.1 ± 0.1	3.1 ± 0.3	4.4 ± 0.3	24.5 ± 2.2	15.7 ± 1.3
Cu	15.7 ± 1.4	2.1 ± 0.2	17.5 ± 1.6	18.3 ± 1.7	20.5 ± 1.9	16.6 ± 1.5	1.3 ± 0.1	1.8 ± 0.2	2.0 ± 0.2	3.4 ± 0.3	18.5 ± 1.4	11.9 ± 0.9
Zn	250.0 ± 25	93 ± 9.0	135.0 ± 12	135.0 ± 13	91.0 ± 9.0	125.0 ± 12	27.0 ± 3.0	165.0 ± 16	55.0 ± 4.0	39 ± 4.0	80 ± 8.0	43.0 ± 4.0
Rb	21.9 ± 0.8	36.6 ± 1.2	31.5 ± 1.1	54.6 ± 1.6	60.9 ± 1.7	31.3 ± 1.1	37.6 ± 1.3	20.4 ± 0.7	52.3 ± 1.5	47.5 ± 1.4	61.9 ± 1.8	26.6 ± 0.9
Sr	23.9 ± 1.5	36.6 ± 2.1	22.5 ± 1.4	44.6 ± 2.7	28.0 ± 1.4	20.5 ± 1.1	23.5 ± 2.0	20.0 ± 1.8	34.7 ± 1.6	36.3 ± 1.8	22.6 ± 1.3	14.5 ± 0.9
Y	180.0 ± 15	101.0 ± 9.0	44.8 ± 4.1	61.6 ± 6.4	25.7 ± 2.3	39.4 ± 3.2	31.2 ± 2.9	32.1 ± 2.6	28.4 ± 2.4	26.1 ± 2.3	40.8 ± 3.5	11.1 ± 0.9
Zr	370.0 ± 28	4.3 ± 0.1	160.0 ± 14	143.0 ± 13	125.0 ± 11	150.0 ± 15	2.4 ± 0.2	2.2 ± 0.2	57.8 ± 4.5	39.9 ± 3.3	110.0 ± 10	55.2 ± 4.4
Cd	0.11 ± 0.02	0.05 ± 0.01	0.12 ± 0.02	0.14 ± 0.02	0.05 ± 0.01	0.11 ± 0.02	0.05 ± 0.01	0.05 ± 0.01	0.05 ± 0.01	0.05 ± 0.01	0.05 ± 0.01	0.05 ± 0.01
Cs	0.8 ± 0.1	6.0 ± 0.5	1.1 ± 0.2	0.8 ± 0.1	3.6 ± 0.3	1.0 ± 0.1	11.1 ± 0.6	5.5 ± 0.4	1.1 ± 0.2	0.6 ± 0.1	1.1 ± 0.2	0.8 ± 0.1
Ba	13.5 ± 1.1	185.0 ± 15	50.0 ± 3.0	107.0 ± 6.0	285.0 ± 7.0	52.3 ± 4.1	181.0 ± 17.0	130.0 ± 7.0	117.0 ± 6.0	92.0 ± 7.0	385.0 ± 19	110.0 ± 7.0
Pb	65.0 ± 8.0	62.0 ± 6.0	49.0 ± 5.0	54.0 ± 5.0	47.4 ± 4.3	43.0 ± 4.2	23.5 ± 1.9	28.5 ± 1.9	23.2 ± 2.1	16.2 ± 1.3	46.2 ± 3.6	19.8 ± 1.7
Th	930 ± 87	830 ± 75	500 ± 49	560 ± 62	200 ± 23	560 ± 63	300 ± 31	370 ± 41	200 ± 25	100 ± 15	37 ± 7.0	35 ± 6.0
U	53.2 ± 4.8	36.3 ± 3.1	15.3 ± 1.8	15.4 ± 1.9	6.7 ± 0.4	11.8 ± 1.7	12.7 ± 1.3	12.4 ± 1.1	5.6 ± 0.3	2.4 ± 0.2	1.7 ± 0.1	1.4 ± 0.1

**Table 3:** Enrichment factor of TEs in soils.

Elements	Kanamana	Basanaputi	Badaputti	Matikhalo	Gopalpur	Aryapalli	Kalyaballi	Jagnyasala	Chhatrapur	Kalipalli	Boxipalli	Venkatraipur
Cr	1.8	1.2	1.8	2.9	2.8	7.7	1.1	1.1	4.2	0.6	1.1	1.5
Mn	2.4	1.1	1.5	1.8	2.2	10.0	0.6	1.1	2.0	1.0	2.2	1.2
Fe	2.5	0.8	1.2	1.8	2.0	8.7	0.5	1.0	2.0	0.7	1.5	0.9
Co	3.0	0.9	1.3	2.1	2.2	9.3	0.5	1.0	1.8	0.7	1.3	0.8
Ni	0.9	0.2	0.2	0.7	0.6	1.6	0.6	1.0	1.8	−0.4	−1.2	−0.3
Cu	1.0	0.2	−0.4	0.9	1.0	1.8	0.6	1.0	2.0	−2.3	−5.5	−2.0
Zn	1.9	0.5	0.9	1.5	1.6	6.3	0.5	1.0	2.0	0.3	1.1	1.6
Rb	0.6	0.8	1.0	0.5	0.9	0.9	0.4	1.0	1.6	0.5	0.6	0.2
Sr	1.1	1.7	1.9	0.9	2.0	2.2	0.6	1.0	2.0	0.8	1.6	0.7
Y	1.2	0.6	0.9	0.9	1.4	7.6	0.2	1.0	1.1	0.6	2.4	0.6
Zr	1.7	0.4	0.7	1.2	1.3	6.5	0.4	1.0	1.9	0.0	0.0	0.0
Cd	1.7	0.1	0.8	1.2	3.5	5.1	0.4	1.0	1.6	0.0	0.0	0.0
Cs	1.0	0.5	1.1	0.8	0.7	1.9	0.5	1.1	4.6	7.4	5.1	3.7
Ba	0.1	0.2	0.4	0.1	0.3	0.0	0.2	1.0	1.2	0.4	0.5	0.3
Pb	1.2	0.4	0.6	0.9	1.1	1.0	0.4	1.1	1.8	0.4	1.3	0.5
Th	15.5	2.7	6.9	13.5	14.0	52.4	0.8	1.0	10.7	6.8	21.7	7.5
U	10.0	1.4	4.0	6.1	8.4	51.3	0.7	1.1	6.7	5.5	20.4	5.4

**Table 4:** Geoaccumulation index values of TEs in soils.

Elements	Kanamana	Basanaputi	Badaputti	Matikhalo	Gopalpur	Aryapalli	Kalyaballi	Jagnyasala	Chhatrapur	Kalipalli	Boxipalli	Venkatraipur
Cr	−1.0	−1.5	−1.4	−0.1	−0.4	0.3	−1.2	−1.9	−0.3	−2.0	−1.5	−0.7
Mn	0.3	−0.9	−0.6	0.2	0.1	1.8	−1.3	−0.5	−0.5	−0.3	0.4	−0.1
Fe	−0.5	−2.3	−1.9	−0.8	−1.1	0.4	−2.5	−1.9	−1.4	−1.8	−1.0	−1.4
Co	0.8	−1.0	−0.6	0.5	0.2	1.6	−1.2	−0.5	−0.5	−0.8	−0.2	−0.5
Ni	−1.9	−4.1	−3.1	−2.1	−2.4	−2.0	−2.2	−1.9	−1.7	0.0	0.0	0.0
Cu	−1.3	−3.8	−2.9	−1.4	−1.4	−1.4	−1.8	−1.4	−1.1	0.0	0.0	0.0
Zn	0.4	−1.6	−0.9	0.3	0.1	1.4	−1.1	−0.3	−0.1	−1.9	−0.1	0.7
Rb	−2.1	−1.4	−1.2	−2.0	−1.1	−3.6	−2.2	−0.7	−1.4	−1.7	−1.8	−2.6
Sr	−4.6	−3.8	−3.6	−4.5	−3.3	−4.4	−5.1	−4.2	−4.3	−4.4	−3.7	−4.6
Y	0.5	−0.5	−0.3	0.3	0.4	2.5	−1.5	0.6	−0.2	0.0	1.7	0.0
Zr	−0.9	−3.1	−2.3	−1.0	−1.5	0.5	−2.5	−1.3	−1.1	−6.9	−6.1	−7.0
Cd	−1.2	0.0	0.0	−2.1	−0.9	0.4	−2.4	−1.6	−1.8	0.0	0.0	0.0
Cs	−2.9	−3.7	−3.1	−2.9	−2.7	−5.1	−2.5	−2.6	−2.1	−0.1	0.6	−0.4
Ba	−4.3	−3.4	−3.2	−4.2	−3.1	0.0	−3.2	−1.0	−2.0	−2.4	−2.3	−2.8
Pb	0.9	−0.7	−0.2	0.7	0.6	0.0	−0.3	0.9	0.9	−0.1	1.3	0.2
Th	5.0	2.2	2.6	5.1	4.3	5.8	1.3	1.5	4.0	4.4	5.7	4.6
U	1.9	−1.1	−0.7	1.4	0.7	3.6	−1.4	−1.2	0.9	1.6	3.2	1.6

**Table 5:** Descriptive statistics of REEs (µg g^−1^) in soils (*n* = 36).

Element	Mean	Min	Median	Max	Skewness	Kurtosis	CV
La	540.76	17.65	334.97	2770.36	1.86	3.67	1.16
Ce	1121.56	40.62	687.72	5797.34	1.90	3.81	1.16
Pr	119.09	3.91	68.66	638.91	1.94	4.09	1.20
Nd	458.58	16.10	269.25	2557.04	2.06	4.78	1.22
Sm	66.88	2.74	41.37	389.59	2.25	6.12	1.22
Eu	1.96	0.31	1.49	7.96	2.06	4.78	0.88
Gd	39.97	1.96	24.16	228.39	2.25	6.05	1.19
Tb	4.23	0.34	2.46	26.18	2.67	8.97	1.21
Dy	13.44	1.40	7.70	89.13	3.32	13.93	1.19
Ho	2.05	0.23	1.23	11.03	2.62	8.22	1.07
Er	5.37	0.64	3.67	32.50	3.23	13.28	1.08
Tm	0.93	0.09	0.51	4.99	2.37	5.71	1.19
Yb	4.30	0.65	3.12	26.32	3.61	16.44	1.05
Lu	0.95	0.10	0.46	5.66	2.50	6.65	1.26
ΣREE	2431.19	101.31	1469.72	12,911.35	1.98	4.32	1.17
ΣLREE	2308.82	85.40	1403.06	12,160.34	1.94	4.04	1.18
ΣHREE	71.24	6.48	44.25	421.50	2.65	8.88	1.14
Eu/Eu*	0.21	0.06	0.11	0.78	1.59	1.39	0.95
Ce/Ce*	1.46	0.98	1.04	4.51	2.51	4.66	0.75
(La/Sm) _N_	5.31	2.87	4.92	10.06	2.29	4.58	0.29
(La/Yb) _N_	84.35	5.40	82.44	211.66	0.28	−1.15	0.70
(Gd/Yb) _N_	7.23	1.27	7.25	18.59	0.39	−0.70	0.64

CV, coefficient variant; Min, minimum; Max, maximum. Eu/Eu* and Ce/Ce* are the calculated europium and cerium anomalies, respectively. Subscript N indicates chondrite normalized values.

**Table 6:** Pearson correlation coefficient of Th, U and REEs in soils (*n* = 36).

	La	Ce	Pr	Nd	Sm	Eu	Gd	Tb	Dy	Ho	Er	Tm	Yb	Lu	Th	U
La	1.00															
Ce	1.00	1.00														
Pr	1.00	1.00	1.00													
Nd	1.00	1.00	1.00	1.00												
Sm	1.00	1.00	1.00	1.00	1.00											
Eu	0.79	0.80	0.79	0.77	0.77	1.00										
Gd	0.99	0.99	1.00	0.99	1.00	0.81	1.00									
Tb	0.98	0.98	0.99	0.99	0.99	0.79	1.00	1.00								
Dy	0.95	0.95	0.95	0.96	0.97	0.75	0.97	0.99	1.00							
Ho	0.88	0.89	0.89	0.87	0.88	0.95	0.91	0.91	0.89	1.00						
Er	0.91	0.91	0.91	0.91	0.93	0.86	0.94	0.96	0.97	0.97	1.00					
Tm	0.67	0.68	0.68	0.64	0.65	0.96	0.70	0.68	0.64	0.92	0.80	1.00				
Yb	0.85	0.85	0.86	0.86	0.88	0.77	0.89	0.92	0.96	0.91	0.98	0.70	1.00			
Lu	0.61	0.62	0.62	0.58	0.58	0.95	0.64	0.61	0.57	0.87	0.74	0.99	0.63	1.00		
Th	0.90	0.90	0.90	0.89	0.87	0.64	0.86	0.83	0.75	0.68	0.69	0.50	0.60	0.46	1.00	
U	0.95	0.95	0.95	0.96	0.97	0.78	0.97	0.99	0.99	0.91	0.96	0.69	0.93	0.62	0.75	1.00

## Data Availability

The data presented in this study are available on request from the corresponding author.

## Sample Availability

Samples are available from the authors.
